# Handcuff use on forensic psychiatry wards

**DOI:** 10.1192/bjo.2021.934

**Published:** 2021-06-18

**Authors:** Rachel Swain, Deborah Klayman, David Reiss, Kruti Buch, Sarah Roberts

**Affiliations:** West London NHS Trust

## Abstract

**Aims:**

This project aimed to assess the use of handcuffs in a secure forensic mental health hospital.

**Background:**

Handcuffs are used by secure forensic psychiatric wards where patients need to leave the ward and require added restrictive measures for their own or other's safety. The decision to use handcuffs is made by the multi-disciplinary team, with the input of the unit's clinical security team and is assessed based on individual risk and need. This study investigated the frequency, duration and purpose of handcuff use in one secure forensic mental health unit, encompassing 8 male medium secure wards, 5 male low secure wards, 1 adolescent secure ward,1 female low secure ward and 5 female medium secure wards.

**Method:**

Handcuff use was recorded contemporaneously by ward staff in a specialised handcuff proforma. This data were then compiled to assess the number of instances of use, the mental health section applicable to the patient, the reason the patient needed to leave the unit, and the duration of use (including the time period for which the handcuffs were removed during the visit, if applicable.) Data from these forms over an 18 month period were analysed.

**Result:**

Over the 18 months, there were a total of 347 uses of handcuffs, with an average of 18.3 occurrences per month. In 55 cases, the patients were detained under a civil section, with the remaining instances occurring in patients detained under forensic section. 47% were unsentenced prisoners.

The most common destination for patients was the general medical hospital, which accounted for 49% of all visits. Court was the second most common destination, with 39% of uses.

The average duration spent in handcuffs was 3.3 hours. The average time that the handcuffs were taken off during the transfer was 1.2 hours.

**Conclusion:**

Through ongoing education and supervision by the clinical security team, handcuff use in this forensic service was limited to essential situations, most often to allow treatment of physical health issues off-site. A large proportion of instances involved unsentenced prisoners and court attendances, where the risk of absconsion might be particularly high. Duration spent in handcuffs was kept to a minimum, with cuffs being removed where possible. The service strives to continue such good practices and to identify further ways to reduce handcuff use, such as using video-conferencing as an alternative court attendance.

